# Preliminary Proof of the Concept of Wild (Feral) Horses Following Light Aircraft into a Trap

**DOI:** 10.3390/ani10010080

**Published:** 2020-01-02

**Authors:** Sue McDonnell, Catherine Torcivia

**Affiliations:** University of Pennsylvania School of Veterinary Medicine New Bolton Center, 382 W Street Road, Kennett Square, Pennsylvania, PA 19382, USA; torcivia@vet.upenn.edu

**Keywords:** *Equus caballus*, drone, unmanned aerial vehicle, gather, muster, capture, trap, low-stress handling

## Abstract

**Simple Summary:**

Our long-term goal is to develop less stressful ways of gathering and handling wild horses for necessary capture, either for permanent removal from the range or for repeated application of fertility control treatments. This report describes preliminary evaluation of the concept of leading wild horses into a corral using light aircraft as a less stressful, less expensive, and safer alternative to the current most common practice of driving horses with helicopters into traps. In a model herd of semi-feral managed ponies, an entire herd was successfully led by a remotely operated quadcopter drone into simulated capture enclosures.

**Abstract:**

Feral horses, wherever managed, typically require population control involving capture for permanent removal or repeatedly for fertility control treatments. The most common method for capturing feral horses is helicopter chasing into traps. With this fear-based strategy, it is difficult to safely capture entire groups. Recapture becomes increasingly difficult, with greater safety risks for pilots and ground staff. As preliminary proof of the concept of capturing free-roaming horses by leading into enclosures with light aircraft rather than driving with helicopters, a consumer-grade quadcopter drone was used to lead a herd of 123 semi-feral ponies into simulated traps. The technique was successful on the first attempt as well as for seven of nine additional attempts over a period of 4 weeks, repeatedly to the same as well as to different destinations. The pace of following was primarily a fast walk, with occasional slow trot. Family integrity was maintained. This work demonstrates preliminary proof of the concept of repeated capture of horses by leading with aircraft rather than chasing. If successfully demonstrated in more extensive rangeland conditions, this method may eventually provide a lower-stress, more repeatable option of capturing feral horses, with implications for improved animal and human safety and welfare.

## 1. Introduction

Free-ranging feral horse (*Equus caballus*) populations exist in a number of countries around the world, including Australia, United States, Canada, Spain, France, Portugal, Brazil, Namibia, Japan, Ethiopia, Portugal, Sri Lanka, Columbia, Wales, Romania, and New Zealand. Most of these populations are managed as wildlife by various government entities. In the United States, the Department of Interior’s Bureau of Land Management is charged with managing feral horses on public lands, officially designated as *wild* in the Wild Horse and Burro Act of 1971 [1]. The feral horses in the Western United States, whether on public, private, or native American tribal lands, are also known as mustangs. Though technically *feral*, as they are derived from once domesticated stock, these and other free-running horse populations around the world are commonly called wild horses [2].

Management of feral horse populations typically requires periodic capture of free-roaming herds (referred to as *gathering* or *mustering*), either for permanent removal from the range, or in recent years for application of population fertility control interventions before return to the range. The most common modern method of gathering feral horses in the United States has involved the use of helicopters to drive herds into well-designed temporary traps. The technique involves approaching the horses in a threatening, though carefully measured, manner that provokes neighboring bands to coalesce as a larger assemblage to “escape” as a group. With skillful directional pressure of their natural escape behavior, groups can be directed by the helicopter into a trap. An example video recording of a helicopter gathering of horses in Australia in 2015 can be viewed online [3]. The United States Department of Interior’s Bureau of Land Management (BLM), which conducts most of the wild horse gathers in the United States, generally considers this capture method humane and the only efficient method for much of the terrain where horses are managed. However, there clearly are challenges and potential welfare threats with this method of capturing horses [4]. While the goal is to move the horses at a reasonably slow pace (trot or slow canter), this is not always achieved. Travelling long distances at any pace faster than a walk and occasional trot may be difficult for foals, pregnant mares, as well as for aged or disabled horses. Individual band members may become separated and left behind on the range. Family band separations can induce panicked attempts to re-unite, resulting in injuries and deaths. Highly aroused groups arriving at speed into small traps can also result in injuries. The percentage of known deaths, including death following traumatic injury while in the trap, has been estimated at approximately 0.5% [5]. Non-lethal injuries are not officially recorded. Additionally, of considerable practical importance, as an open plain-grazing species, horses are efficient learners relative to negative or stressful experiences, and so repeat helicopter-driven capture becomes increasingly difficult [4].

An alternative method of gathering is passive trapping, using, for example, water during droughts, highly palatable feed stuffs, or salt/mineral licks. Passive trapping has the advantage of reduced risks of physical stress and/or injury, social disruption, and traumatic negative first experience with humans that is no doubt deleterious to future capture and/or for those animals removed from the range for eventual taming for domestic care and use. Passive trapping has been used by BLM to a limited extent, more often during draught emergency, with only a few locations where bait trapping is currently expected to be used routinely [6].

The purpose of the work reported here was to explore preliminary proof of the concept of an alternative ethology-informed method of active trapping, using a light aircraft to lead, rather than to drive, bands or herds of free-roaming horses into trap enclosures. This concept is based on the natural instinctive behavioral tendency of horses to become alert to intruders or novel objects and to respond as a herd according to the level of sympathetic arousal evoked. Their behavioral response to novel/potentially threatening stimuli ranges from positive curious interest, to mild concern/vigilant monitoring, or to retreat, either measured or in full panic flight. We hypothesize that a low-flying aircraft, piloted in a manner that evokes and maintains sympathetic arousal within the *curious interest* to *mild concern* level can lead an entire group of free-roaming horses from one area to another and into traps. We further hypothesize that this method will (a) not require any pre-conditioning of the animals; (b) be repeatable as necessary; (c) be controllable to a pace within the range of a fast-walk to occasional easy trot; and (d) will allow family groups to be kept together en route and during capture. Additionally, we hypothesize that stallions will take the lead and, once committed to monitor the aircraft, will call back to alert the remainder of the herd to instinctively follow together. As a preliminary proof of the concept, our model approach was to employ a small consumer-grade quadcopter unmanned aerial vehicle (drone) as the novel mildly threatening stimulus to lead a herd of semi-feral ponies into various simulated traps.

## 2. Materials and Methods

### 2.1. Study Population and Enclosure

The study group consisted of a herd of semi-feral Shetland-type ponies kept at the University of Pennsylvania’s School of Veterinary Medicine’s New Bolton Center large animal campus in Southern Chester County, Pennsylvania. This herd was established in 1994 as a model for the study of reproductive physiology and behavior of horses living continuously under natural social and environmental conditions. This is a closed herd of intact animals (permanent removals for population management, no returns, and no additions other than births). At the time of this work, the herd of 123 animals consisted of ten harem bands, three distinct bachelor bands, and two mixed sex bands of young stallions and fillies in transition from their natal bands. Their 40 acre enclosure includes multiple natural water sources and ample natural forage and browse except in winter when supplemental grass hay is distributed throughout the enclosure. Handling is limited to periodic routine health care (annual vaccination, deworming). This is accomplished by luring (similar to bait trapping) the entire harem or bachelor bands, one band at a time, into a catch pen system with multiple sub-compartments and chutes to enable separation as needed for safe low-stress interaction with individual animals. In addition to luring, animals are reinforced with highly palatable feed treats or mutual grooming style scratching for entering individual treatment pens as well as for complying with mildly aversive health care and research sampling procedures.

### 2.2. Animal Care and Use Approval

The care of these animals and the procedures reported here have been approved by the University of Pennsylvania Animal Care and Use Committee, following all applicable guidelines set forth for research and teaching horses.

### 2.3. Simulated Traps

For the purposes of this study, three small enclosures (¼, ¾, and 1 acre; contiguous to the herds’ permanent enclosure) were available to use as simulated trap destinations. One of these enclosures was adjacent to an area of their enclosure near a swine production facility. This herd historically had avoided that area, especially when in certain weather conditions the swine odor was particularly pungent. This “trap” was included purposefully to simulate a particularly challenging destination.

### 2.4. Drone

A consumer-grade quadcopter drone was used (DJI Phantom 3, Standard model; Shenzhen Dajiang Baiwang Technology Co., Ltd., Shenzhen, China). This model has features recommended for ease of handling for beginner drone pilots, including automatic stabilization and return to base upon request or automatically before battery depletion. It carries a video camera/recorder with the ability to transmit real-time video to a cell phone as an aid to piloting when the drone is out of direct view. In order to maintain a video view of the herd whether approaching or retreating, the pilot learned to fly the drone both forwards when approaching the herd and backwards when retreating.

### 2.5. Personnel

All simulated gather attempts were done by the two authors. One (Catherine Torcivia) did all the piloting of the drone. She was a recent graduate veterinarian with several years’ experience training horses, but no previous experience piloting a drone or leading horses using this ethology-based method. Preparation included 5 training flights in an open field as well as orientation to the behavioral responses of horses corresponding to the level of sympathetic arousal to induce a following response. The other author (Sue McDonnell) served as an assistant. She is an applied animal ethologist and behavioral physiologist, with greater than 35 years’ experience with herding and leading unhandled/untrained horses and ponies, as well as similar work with sheep, cattle, and camelids for repeated low-stress capture or herd relocation. The role of the assistant was to observe the herd in order to instantaneously advise the drone pilot via cell phone concerning the behavioral response of the ponies and suggest drone maneuvers to maintain the herd’s interest and following behavior. She also video recorded the gather process from the ground as feasible, including the drone as it approached the herd and the herd’s response. The assistant was positioned at a distance of at least 50 m from the nearest herd member, either outside or inside the enclosure. This herd is generally accustomed to the routine presence of 1 to 3 field behavior observers similarly standing off at distance of 50 to 100 m for hours at a time, either inside or outside of their enclosure, such that ongoing herd activities appear undisturbed. For several weeks before the start and continuing through this project, behavior observers (including at times the pilot and assistant) were present during most daylight hours other than during these gather sessions.

### 2.6. Simulated Gather Attempts

The proposed plan was to conduct drone gather attempts on up to 10 occasions at 1 to 5 day intervals, weather permitting. The particular model of quadcopter employed is self-stabilizing in wind speeds up to 30 mph, however both smoothness of flight and battery life are markedly reduced in wind speeds greater than 5 mph. Therefore, gather sessions were done only with no more than a light breeze. On any given flight day, the location of the herd was assessed, and a destination and trap planned.

### 2.7. Technique

Behaviors consistent with sympathetic response to a novel, potentially threatening stimulus, at the level that evokes a state of curious alert/mild concern resulting in following (monitoring) typically include alert posture, conflict behaviors, such as sudden outburst of inter-male aggression, rotational head shaking, head threats toward the stimulus, and moderate alarm calls among herd mates [7]. Response of horses to an inanimate remotely controlled ground vehicle has been reported previously, although in round pen training with one horse that included initially chasing before retreating [8].

### 2.8. Flight Data

Drone and ground camera video recordings were reviewed to summarize the number of approaches, the latency (flight time) to evoke to whole herd following, total flight time to the pre-determined destination, as well as gait/s at which the herd followed the drone.

## 3. Results

Drone gather attempts were undertaken on 10 different occasions between June 21 and July 17, 2017. The gather plan and results for each are summarized in Table 1. For eight of the 10 attempts, all 123 animals in the herd reached the planned destination with total flying times ranging from approximately 4 to 38 min flying time. For the trap enclosure near the swine facility, which was expected to be the most challenging destination, successful simulated gathering of the entire herd was accomplished with a flying time of 20 min. The majority of flying time involved initiating following; once the whole herd was following, there was uninterrupted travel into the trap. For one of the two unsuccessful occasions, the alert and whole herd following responses were successfully initiated, but the designated destination was not reached. For the other, momentary following by a few lead ponies was repeatedly initiated, but following by the entire herd was not successfully evoked.

In all cases, one or more stallions were the first to alert to the approach of the drone as well as to initiate following of the drone’s retreat. Those stallions vocalized in a characteristic loud distant call back to the remainder of the herd, which then reflexively coalesced and followed en masse. During travel to the trap, stallions periodically called back, which resulted in re-energizing their focused following. By adjusting the airspeed of the drone, the pace of the herd in general was controllable to a fast walk with occasional slow trot. Typically, one or more juvenile stragglers, that at the time had been either in recumbent sleep or away from their natal band with playmates, cantered or galloped short distances to catch up to their family. As the herd travelled and arrived into the traps, in all cases, harem band integrity was maintained.

After the first few gathers, there was some indication that the ponies habituated to the approach of the drone. Serendipitously, we found that landing at a distance of approximately 15–30 m ahead of the leading animals stimulated renewed curiosity, approach, and then following upon drone take off. For subsequent gather attempts, alteration of the visual/auditory characteristics of the drone by attaching reflective Mylar ribbon streamers to the landing gear also appeared to renew sufficient interest. Additionally, approaching different harem or bachelor groups that had not previously led the herd response was effective.

Typical drone altitude to effectively evoke appropriate level of interest was usually between 2 and 6 m above ground at a distance typically of under 10 m ahead of the leading animals, with a slow gradual approach with intermittent momentary hovering, and faster sweeping retreats of 5 to 10 m. Variation in response seemed related to ambient noise and wind direction, as the propeller sound often appeared to be the initial stimulus attracting attention.

## 4. Discussion

These results provide preliminary proof of the concept that aircraft can effectively lead, as opposed to drive, herds of horses into a trap. Using a small quadcopter drone, the method was successful on the first attempt and was repeatable on multiple occasions in one semi-feral herd. This included leading of the same entire herd to various pre-selected destinations, with some destinations repeated on subsequent occasions. The pace of the herd was controllable to no more than a slow trot, with occasional individual animal exception, and harem band integrity was maintained through the process. Compared to current helicopter chase methods, all of these aspects would likely improve the welfare of the animals during the capture process. This less stressful experience associated with capture would have considerable implications for those horses removed from the range with the goal of adoption into private care, taming, and training.

As hypothesized, leadership of the herd in these gather attempts was primarily by stallions. This may represent an important practical advantage for repeated capture of wild horses for on-range population control. Wild horse population fertility control methods currently in use (immunocontraceptives Porcine zona pellucida and gonadotropin releasing hormone vaccines) require repeated capture and handling. Mares are the target of the most aversive handling, that would likely increase difficulty for future capture. If band integrity can be maintained, the experience for stallions will be even less negative than current practice. Additionally, if all captured horses could be positively reinforced upon entering the trap and during capture (i.e., fed highly palatable grain or hay), that would help to offset the negative aspects of the capture. If that were the case, repeated capture would be expected to be more efficient and less problematic. With repeated exposure, the aircraft would likely become a conditioned positive stimulus, just as a ranch vehicle that is associated with positive experience (typically feeding) becomes a conditioned positive stimulus for horses or cattle. With these semi-feral ponies, no grain or hay was provided as positive reinforcement, either along the routes or at the destinations.

In this work, it appeared that ponies habituated to the drone as a novel mildly threatening stimulus. When we altered the appearance and sound, interest was renewed. We propose that, if associated with a positive outcome, the motivation for following the drone would change from the monitoring of a potential threat to positive and goal-directed—in which case, habituation to the stimulus would not be problematic. Rather the strength of following response should increase with continued positive experience. This would make repeated gathering an even more efficient, less stressful, and safer experience. The response of horses to inanimate stimuli, including behavior modification using positive and negative reinforcement, has been demonstrated in a round pen ground training paradigm, using a remote controlled toy car [8]. The addition of positive reinforcement in the gather context is worthy of further study. Provision of small caches of highly palliative feed stuffs along the route of travel would theoretically increase positive motivation for following the aircraft.

There are obvious limitations to this semi-feral herd and its environment as a model for truly feral horses. For example, the distance traveled on any occasion was limited, and considerably less than is done with helicopter gathers, which can be up to 16 km. It remains to be tested how far over more difficult terrain a herd of horses will maintain following. It is also reasonable to question whether this semi-feral herd, that is much more accustomed to people, as well as living in a familiar enclosure adjacent to the simulated traps, is an adequate model for demonstration of the induced following phenomenon. It is important to appreciate that the following behavior was induced by the drone, and not associated with people who were remote to the drone and to the herd. The drone was a completely novel and unusual stimulus, presumably ruling out previous experience with humans as a factor. This would be the case in an open-range application, where the remote pilot would be well away from the herd as it travels and not near the trap. Trap gates also could be closed by remote control, eliminating the need for people at the trap during capture.

Our initial aim was to use a drone simply as a model for manned light aircraft. Currently, manned aircraft minimum airspeeds, even for ultralight and auto-gyro craft, would require carefully flying a pattern, for example zig-zag, that would maintain a stimulus to effectively lead a herd at the desired slow pace. At the current pace of technological advances in unmanned aerial vehicles, it is expected that drones may be more effective and safer than manned aircraft.

At the start of this project, the drone pilot was inexperienced with drone technology, as well as with piloting aircraft of any type. She was well familiar with domestically managed horses, but not specifically with the following behavior phenomenon or with horses living in semi-feral natural social and environmental conditions. Nonetheless, with minimal orientation and real-time remote guidance of the equine ethologist experienced with using this behavioral phenomenon to lead horses on foot, the drone pilot’s first attempt was successful. After only a few sessions, this pilot became proficient at flying the drone backwards in order to use the camera to monitor the leader animals’ behavior in order to make instantaneous flight path and speed adjustments required to maintain the herd’s appropriate level of interest. This rapid acquisition of behavior observation and piloting skills is encouraging for training of additional drone pilots. Further improvements in technology, such as both rear- and forward-facing drone cameras and a viewing device with larger screen (than cell phone), would likely enhance acquisition of those skills. In practice, the expectation would be to become proficient in these skills in advance of an actual gather attempt by practicing in simulated large pasture herds.

For this preliminary work, we chose to use a small commercially available drone for several reasons. These reasons included cost and features designed to enhance success of novice drone pilots. Much more sophisticated drone technology would be required for gathering under most range conditions of various wild horse herd management areas. Long battery life for the expected greater distances, as well a greater range from the control base, and advanced on-board real time video camera for simultaneous forward and rear view real-time monitoring of herd and the route of travel. The drone pilot would also typically require advance knowledge of the terrain and best route of travel both for the horses and for the drone. However, the technology is available (at a much greater cost) that can meet these requirements.

Drones are currently in use for herding domestic animals, including sheep and cattle [9]. Anecdotally, we have heard of plans to use drones to move wildlife, including horses. The strategy is to use the traditional driving method of pressure from behind, as opposed to leading as we did with these ponies. Drones are also being employed in other aspects of wildlife management and research, for example for performing population surveys and studying behavior or movement patterns [10,11].

Finally, the authors plan to next obtain further proof of the concept with a herd of feral horses that have been removed from the range and are now privately managed in a refuge. The enclosure is far larger than what was available with this semi-feral herd of ponies, and the terrain is more typical of some of the wild horse management areas. The proposed herd includes intact stallions, mares, and young living relatively undisturbed under natural social conditions. Should this method continue to show promise, further research could include obtaining data on physiologic and behavioral measures of stress for comparison with other methods of capture.

## 5. Conclusions

This preliminary proof of the concept project in one model herd demonstrates that horses can be led using a drone. The technique was effective on the initial attempt and repeatedly over a period of one month. Further work will be required to evaluate the feasibility of this method in truly feral herds, using more sophisticated drone technology suitable for longer flights, with more sophisticated onboard video for remotely locating and moving herds on expansive terrain.

## Figures and Tables

**Table 1:** Summary of drone gathers.

Date	Intended Destination/Miles From Starting Point	Approaches/Minutes to Whole Herd Following	Total Flight Duration (Minutes)	Gaits *
Jun 21	Trap A/0.3	9/4.5	6.3	ST-FT (SCS)
Jun 22	Trap B/0.4	1/0	4.25	ST-FT (SCS)
Jun 23	Trap C/0.3	13/8	20.1	FW-ST-SC
Jun 26	Trap A/0.3	23/3 unsuccessful **		
Jun 27	Trap A/0.5	11/16 unsuccessful ***		
Jul 11	Swamp/0.25	29/10	32	FW-ST-SC
Jul 12	Trap A/0.3	1/0.6	10.5	SW-FW (SCS)
Jul 12	Trap A/0.3	26/22	24.5	SW-FW-ST
Jul 14	Trap B/0.3	2/1	7.2	ST-FT-SC
Jul 17	Trap B/0.4	41/10.5	38	SW

* Gaits: SW = slow walk; FW = fast walk; ST = slow trot; FT = fast trot; SC = slow canter; (SCS) = straggler juveniles slow canter to catch up to family band. ** Wind conditions marginal for auto stabilization capacity, causing rapid battery drain, requiring battery change. *** Low foliage over narrow passage limited drone path of flight.
